# Single-cell analysis reveals TLR-induced macrophage heterogeneity and quorum sensing dictate population wide anti-inflammatory feedback in response to LPS

**DOI:** 10.3389/fimmu.2023.1135223

**Published:** 2023-02-24

**Authors:** Bart M. Tiemeijer, Sebastiaan Heester, Ashley Y. W. Sturtewagen, Anthal I. P. M. Smits, Jurjen Tel

**Affiliations:** ^1^ Laboratory of Immunoengineering, Department of Biomedical Engineering, Eindhoven University of Technology, Eindhoven, Netherlands; ^2^ Institute for Complex Molecular Systems, Eindhoven University of Technology, Eindhoven, Netherlands; ^3^ Laboratory of Soft Tissue Engineering and Mechanobiology, Department Biomedical Engineering, Eindhoven University of Technology, Eindhoven, Netherlands

**Keywords:** single-cell, macrophage, TLR4, IL-10, heterogeneity

## Abstract

The role of macrophages in controlling tissue inflammation is indispensable to ensure a context-appropriate response to pathogens whilst preventing excessive tissue damage. Their initial response is largely characterized by high production of tumor necrosis factor alpha (TNFα) which primes and attracts other immune cells, thereafter, followed by production of interleukin 10 (IL-10) which inhibits cell activation and steers towards resolving of inflammation. This delicate balance is understood at a population level but how it is initiated at a single-cell level remains elusive. Here, we utilize our previously developed droplet approach to probe single-cell macrophage activation in response to toll-like receptor 4 (TLR4) stimulation, and how single-cell heterogeneity and cellular communication affect macrophage-mediated inflammatory homeostasis. We show that only a fraction of macrophages can produce IL-10 in addition to TNFα upon LPS-induced activation, and that these cells are not phenotypically different from IL-10 non-producers nor exhibit a distinct transcriptional pathway. Finally, we demonstrate that the dynamics of TNFα and IL-10 are heavily controlled by macrophage density as evidenced by 3D hydrogel cultures suggesting a potential role for quorum sensing. These exploratory results emphasize the relevance of understanding the complex communication between macrophages and other immune cells and how these amount to population-wide responses.

## Introduction

When facing external cues, the cells of the immune system regulate their combined responses by tight communication. To do so, a large array of paracrine factors is produced including chemokines, pro-, and anti-inflammatory cytokines (1). These factors dictate the decision of cells to partake in the immune response, thus maintaining immune homeostasis balancing between excessive or inadequate inflammation. Macrophages are tissue-resident cells which play a dominant role in the early response to invading microbes by rapid production of pro-inflammatory factors such as tumor necrosis factor alpha (TNFα) (2). TNFα is one of the first cytokines that is secreted in response to infection, thereby sounding the alarm as a first step to protect the tissue from danger (3). It is produced in response to toll-like receptor (TLR) stimulation such as activation of TLR4 by the microbial component lipopolysaccharide (LPS) (4, 5). Macrophages are specialized in neutralizing various types and doses of pathogens whilst minimizing excessive and damaging inflammation (1, 6). To control this response, they are equipped with the ability to produce the anti-inflammatory cytokine interleukin 10 (IL-10) (7, 8). IL-10 is a very potent inhibitor of inflammation which targets innate immunity by reducing cytokine and chemokine production (9) and limiting production of microbicidal agents such as nitric oxide and reactive oxygen species (10). Additionally, it dampens the adaptive immune response by downregulation of major histocompatibility complexes (11) and costimulatory and adhesion molecules (12). Thus, by controlling both the “gas” and “break” of early tissue inflammatory response, macrophages are key players in orchestrating a context-appropriate response to microbial invasion (13–15).

Due to their dominant pro- and anti-inflammatory roles TNFα and IL-10 are generally regarded as both the major regulators as well as reliable indicators of inflammatory homeostasis (16–19). With TLR4 activation resulting in both TNFα and IL-10 secretion and the two having various modes of interaction with each other (20–22), their dynamics make for an intriguing but still not thoroughly understood interplay. Macrophage populations are heterogenous by nature (23) and have been observed to display a wide range of cytokine signatures (18), which in turn affects their decision for pro- or anti-inflammatory tendency (24). This functional plasticity combined with potent cytokine production makes the macrophage compartment highly dynamic (25, 26). Additionally, their cytokine-mediated communication is dependent on cellular density (27, 28), cellular pre-disposition (29, 30), and molecular gradients (31), making it a highly complex system. Apart from microbial infection, macrophage-mediated immune homeostasis plays an important role in wound healing (32, 33), the onset of atherosclerosis (34, 35), tissue engineering approaches (36, 37) and the tumor microenvironment (38, 39). Although these instances are very different from the model of TLR4-mediated inflammation, investigating the fundamentals of macrophage communication during inflammation could improve the understanding of such diseases. Therefore, understanding the exact mechanisms underlying single-cell macrophage heterogeneity and its regulatory role in scaling the local immune response is indispensable for the development of therapeutics targeting dysregulation of macrophage-mediated inflammatory homeostasis.

Here, we investigate the heterogeneity of macrophage cytokine secretion using droplet-based microfluidics which is a commonly used tool for single-cell approaches (40–42). Our platform allows for stimulation, cultivation, and interrogation of individual immune cells, revealing divergent functionalities which would otherwise have been masked in the averaged measurement of bulk populations (43–45). We focus on the ability of individual macrophages to maintain immunologic homeostasis based on secretion of the two key cytokines, TNFα and IL-10. Furthermore, we investigate how the observed heterogeneity is affected by cellular communication and cell density. Improved knowledge over these complex cytokine signaling networks can hold novel therapeutic potential targeting macrophage-related inflammatory imbalance and chronic inflammation.

## Materials and methods

### Monocyte isolation and macrophages differentiation

Using Lymphoprep density gradient (Stemcell technologies) peripheral blood mononuclear cells were isolated from buffy coats obtained from healthy human donors (Sanquin bloodbank), after written informed consent per the Declaration of Helsinki and according to the institutional guidelines. From these cells monocytes were isolated either using adherence or magnetic associated cell sorting (Pan monocyte isolation kit, Miltenyi Biotec). The cells were subsequently plated in Nunc™ culture flasks (Thermofisher) at 7 × 10^5^ cells/cm^2^ in Roswell Park Memorial Institute (RPMI) 1640 (Gibco, life technologies) + 2% human serum (HS, Sanquin blood bank) + 1% Penicillin streptomycin (Gibco, life technologies), referred to after this as culture medium. The culture was supplemented with 20 ng/ml macrophage colony-stimulating factor (M-CSF) (Peprotech) to differentiate monocytes into macrophages over the course of five days, with a media change on day three. On day five cells were washed with PBS and detached using Accutase (Stemcell technologies).

### Macrophage stimulation and receptor blocking

On day five, after cell detachment, macrophages were seeded in culture medium without M-CSF, but supplemented with the appropriate stimuli in the following concentrations: 100 ng/ml lipopolysaccharide (LPS, from *Escherichia coli*, Merck), 5 µg/ml Resiquimod (R848, Enzo life sciences), 100 ng/ml Pam3CSK4 (Biotechne), 5 µg/ml Polyinosinic:polycytidylic acid (poly I:C, *In vivo*gen), 5 µg/ml CPG-c (Enzo Life sciences), 100 ng/ml interferon-gamma (IFNγ, Peprotech), 40 ng/ml interleukin-4 (IL-4, Peprotech), 20 ng/ml interleukin-13 (IL-13, Peprotech), 50 U/ml interferon beta (IFNβ, Peprotech), 20 ng/ml transforming growth factor beta (TGFβ, *In vivo*Gen), 100 ng/ml tumor necrosis factor alpha (TNFα, Peprotech), 100 pg/ml interleukin-10 (IL-10, Peprotech). Due to the volume-per-cell difference between bulk and droplet cultures, the concentrations of stimuli in droplets were adjusted to keep the absolute amount of stimuli-per-cell constant. Il-10 receptor blocking antibodies (αIL-10R, R&D systems), were added to cultures 30 minutes prior to any other stimuli at 6 µg/ml. Cytokine neutralizing antibodies were added during stimulation at the following concentrations: 400 ng/ml IFNβ neutralizing antibody (Biotechne), 100 µg/ml TNFα neutralizing antibody (Adalimumab). Trichostatin A (Sigma Aldrich) was added 30 minutes prior to other stimuli at 1 µg/ml.

### Fabrication of microfluidic droplet devices

Devices were produced using soft photolithography for which the photomasks were ordered from CAD/Art Services, Inc. (Bandon, Oregon). The PDMS molds were made on silicon wafers by spin coating with SU-8 3000 or SU-8 3050 photoresist (both Microresist Technologies) to obtain 30 or 70 µm of channel height, respectively. Our 3-inlet PDMS devices were fabricated by pouring a 10:1 mix of SYLGARD^®^ 184 PDMS and SYLGARD^®^ 184 curing agent (both from Merck) onto the silicon wafers and curing for 2 hours at 65°C. Using a 1.2 mm biopsy puncher holes for the inlets and outlet were punched. The devices were bonded to glass slides using a plasma asher (Emitech, K1050X). After which, the channels were treated with 5% perfluorooctyltriethoxysilane in HFE-7500 fluorinated oil (both from Fluorochem), to make the channels hydrophobic. After 1 hour incubation at 65°C, and flushing with HFE-7500, the devices were incubated overnight at 65°C for thermal bonding and drying.

### Droplet production, cell encapsulation and cell retrieval

Droplets were produced using our previously published tip-loading platform (46). In short, cell and cytokine solutions were drawn into a 200 μl pipet tip and loaded onto the inlets in the PDMS chip. The pipet tip unloading was performed by a neMESYS microfluidic pump (Cetoni). HFE oil containing 2.5% PicoSurf (SphereFluidics) was used as the continuous phase at a flowrate of 30 μl/min for single-cell droplets and 10 μl/min for multi-cell droplets, while cells and the cytokines were flowed at a speed of 5 μl/min for single-cell droplets and 1.6 μl/min for multi-cell droplets. Cell concentration at the droplet formation point was 2×10^6^ cells/ml for single-cell droplets and 2.5×10^6^ (~2 cells/droplet), 5×10^6^ (~4 cells/droplet), 10×10^6^ (~8 cells/droplet), and 15×10^6^ cells/ml (~12 cells/droplet) for the multi-cell droplets. During production, 2 µl of droplet suspension was imaged for each condition using an EVOS™ microscope (ThermoFisher scientific). To verify consistent droplet size and cell distribution these images were checked by measuring droplet diameter using ImageJ software. Droplets were collected in an Eppendorf tube from the outlet, and 150 µl of culture medium was added on top of the emulsions to prevent evaporation of HFE oil. Incubation of droplets was performed for 24 h at 37°C and 5% CO2, after which droplets were de-emulsified by addition of 20% 1H,1H,2H,2H-perfluoro-1-octanol (PFO) in HFE-7500 at a 1:1 volume ratio. After de-emulsification the upper interface of medium containing cells could be collected and processed for further analysis.

### Cell staining and flow cytometric analysis

For detection of cytokine secretion cells were coated with capture antibodies for IL-10 and TNFα (Miltenyi Biotec) prior to macrophage stimulation. After stimulation cells from bulk or droplet cultures were collected and stained with Zombie NIR™ (Biolegend) at a final dilution of 1:2.000 to check viability. After washing cells were stained using an antibody cocktail of the following fluorescent antibodies: anti-Human cluster of differentiation 80 (CD80)- APC-R700 (BD Bioscience), anti-Human C-C chemokine receptor 7 (CCR7) - Brilliant violet 421™, anti-Human CD206- PE/Cy7, anti-Human CD200R- PE/Dazzle™ 594, (all from Biolegend), anti-human TNFα-APC and anti-human IL-10-PE (both from Miltenyi Biotec). Flow cytometric analysis was performed using FACScanto II or FACSaria III (both from BD bioscience). Results were analyzed using FlowJo (FlowJo LLC). Marker expression was quantified using median fluorescent intensity (MFI).

### Flow cytometry based single-cell cytokine quantification using cytokine titration

Macrophages were coated with TNFα, and IL-10 capture antibodies and split into samples of 100.000 cells to be incubated with both recombinant IL-10 and TNFα at varying concentrations. Thereafter all samples were stained using TNFα and IL-10 specific fluorescent antibodies. After flow cytometric measurement the MFI of each sample was matched with the corresponding cytokine concentration to create a titration curve. Using the obtained curve, single-cell fluorescent values could be directly converted to single-cell cytokine content.

### Enzyme-linked immunosorbent assay-based (ELISA) cytokine quantification

Media samples recovered from stimulated macrophages were spun down at 10.000 RPM for 10 minutes to ensure cell-free samples, and afterwards stored at -20°C until measurement. For droplet samples the exact volume of media and corresponding cell number injected into the droplets was registered to allow normalization to volume and cell count and subsequent comparison to bulk cultures. Quantification of cytokine production was performed using TNFα and IL-10 ELISA kits (ELISA MAX Deluxe, Biolegend) according to manufacturer’s protocols. Data was processed using Prism9 (Graphpad software).

### Cytokine production-based sorting and sequencing

Macrophages were coated with TNFα and IL-10 specific capture antibodies and cultured in droplets with LPS for either 2, 4 or 8 hours. After de-emulsification the cells were stained for viability, and cell-bound TNFα and IL-10. Viable cells were sorted into TNFα^+^/IL10^-^ and TNFα^+^/IL-10^+^ populations for each timepoint, using FACSaria III (BD bioscience). Additionally, an extra sample of unstimulated cells was sorted as a control population for each donor. After sorting, cells were spun down and cell-pellets were resuspended in RLT buffer (Qiagen RNeasy kit) for lysis, and flash frozen at -80°C for storage until mRNA isolation. To minimize sample variation mRNA was isolated from all donors simultaneously using a RNeasy kit (Qiagen), which utilizes silica-membrane binding to purify and ensure stability of mRNA. Isolated mRNA was flash frozen and sent to Genomscan (Leiden, The Netherlands) for quality control, sequencing, and data processing. Quality control was performed using Fragment analyzer (Advanced Analytical Technologies), sequencing was performed using Illumina NovaSeq 6000 (Illumina), and data analysis was performed using their standard operating procedure which includes; library preparation quality control, raw data quality control, processing of unique molecular identifiers, adapter trimming, alignment of short reads, feature counting, and differential expression analysis. PCA analysis and differential expression for replicate samples was performed using DESeq2 v2-1.14 package (47), whilst single-sample comparisons were performed using DESeq v1.5 (48). For further analysis the output of the differential expression analyses was used from which all additional plots were generated. All p-values were adjusted for false discovery rate.

### Agarose encapsulation for cell density experiments

4% w/v ultra-low melting point agarose (Merck) was dissolved in RPMI medium at 75°C overnight and cooled down to 37°C, after which 400 ng/ml LPS in culture media was added in a 1:1 ratio. Macrophages in culture media were brought to 37°C as well and mixed with the agarose + LPS mixture in a 1:1 ratio to obtain 1% w/v agarose containing 100 ng/ml LPS and cells at 2.5×10 (6), 5×10 (6), 10×10 (6), or 15×10 (6) cells/ml. Immediately after, the cell solutions were added to a 4°C wells-plate to ensure instantaneous agarose gelation and homogenous cell distribution throughout the gels. After 2, 4, 8, or 24 hours an identical volume of media was added on top of the gels and agitated for 15 minutes to let the cell-produced cytokines diffuse from the gel into the media, which was then aspirated and processed for ELISA quantification of TNFα and IL-10.

### Multi-cell droplet model

To predict the fraction of IL-10 positive cells in multi-cell droplets, a model was written which calculates the chance of an IL-10 producer being present in a droplet and how many other cells this would make positive as well. The model considers the possibilities for every droplet content between 0 cells/droplet and 30 cells/droplet based on Poisson statistics calculated from cell concentration and droplet size, and uses the fraction of IL-10 secreting cells as obtained from single-cell experiments:


$$Fraction\;of\;IL10^{+}cells=\;\frac{\sum_{k=1}^{30}\frac{a^{k}*e^{-a}}{k!}b*k^{2}}{\sum_{k=1}^{30}\frac{a^{k}*e^{-a}}{k!}*k}*100$$

Where *a* is the average number of cells per droplet, *e* represents Euler`s number (=2.71828…), and *b* represents the fraction of IL-10 producers as determined from single-cell experiments.

### Statistical analysis

Data are shown as mean ± standard error of the mean (SEM) unless indicated differently. Statistical analysis was performed using Prism9 (Graphpad software) using tests indicated in figure legends, and if no significance is mentioned then no significance was found. p< 0.05 was considered significant.

## Results and discussion

### IL-10 mediated anti-inflammatory feedback is regulated by a small subset of macrophages

**Figure 1:**
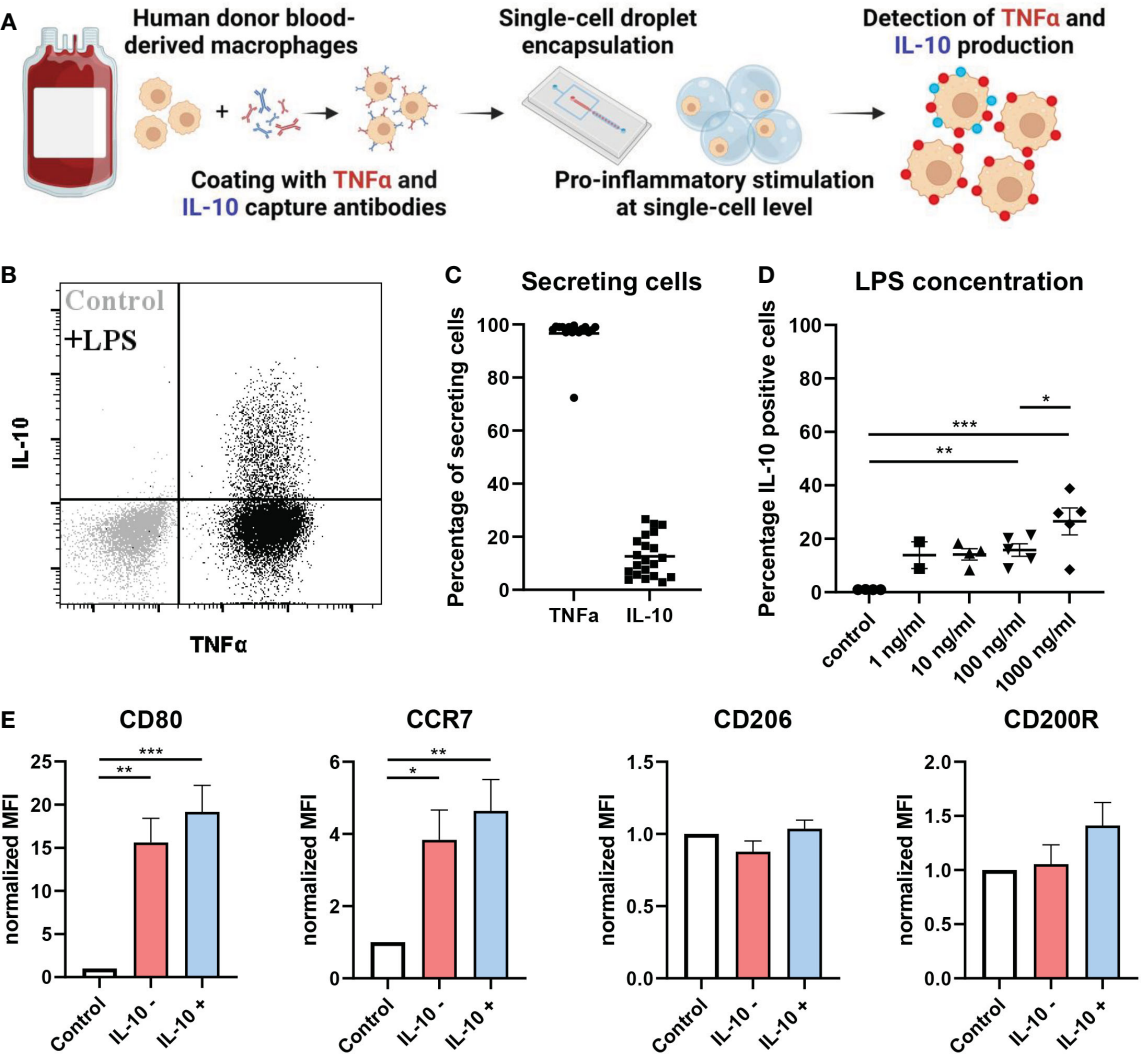
At single-cell level IL-10 is produced by a small population of macrophages. **(A)** Schematic of workflow. **(B)** flow cytometric analysis of TNFα and IL-10 secretion by unstimulated control (grey) and LPS stimulated (black) macrophages in single-cell droplet culture from one representative donor. **(C)** Percentage of cells producing either TNFα or IL-10 after LPS stimulation at single-cell level (n=21 independent donors). **(D)** The effect of LPS concentration on fraction of IL-10 producing cells for n=2 to 5 independent donors. Significance was tested using one-way ANOVA and *post-hoc* Tukey test with * p<0.05, **p<0.01, and ***p<0.001. **(E)** Flow cytometric analysis of pro-inflammatory markers (CD80 and CCR7) and anti-inflammatory markers (CD206 and CD200R) in control condition or after LPS stimulation for both IL-10 producers and non-producers of n=4 independent donors. Significance was tested using one-way ANOVA and *post-hoc* Tukey test with * p<0.05, **p<0.01, and ***p<0.001.

Previously, we and others have described phenotypic heterogeneity within the macrophage compartment (49–52). Here, we investigate how single macrophages integrate pathogenic triggers to generate IL-10 and TNFα responses. To that end human macrophages were stimulated both in bulk and individually encapsulated in droplets using our droplet-based microfluidic platform (**Figure 1A**, **Supplementary Figures 1**, **2**). Cells were activated by the common bacterial antigen lipopolysaccharide (LPS) which is detected by the TLR4 receptor. By providing LPS *via* a separate inlet on the microfluidic chip we ensured that single macrophages only received the stimulating agent upon encapsulation, preventing that juxtacrine- or paracrine interactions influence the cellular response (**Supplementary Figure 1A**). Remarkably, we observed that under single-cell stimulatory conditions only a small percentage (~10%) of cells secreted IL-10, whereas all cells secreted TNFα (**Figures 1B, C**). We encapsulated cells with varying concentrations of LPS and measured the fraction of cells producing IL10 (**Figure 1D**). Surprisingly, we only observed minor variations in the fraction of macrophages secreting IL-10 irrespective of LPS concentrations within a biologically relevant range, implying a robust potentially predetermined mechanism. Proper macrophage activation was verified by studying M1-associated marker expression of CD80 and C-C chemokine receptor type 7 (CCR7) and M2-associated anti-inflammatory markers CD206 and CD200-receptor (CD200R). As expected, macrophages upregulated CD80 and CCR7 expression upon LPS stimulation with minimal levels of CD206 and CD200R (**Figure 1E**). Interestingly, no significant phenotypical difference was found between IL-10 positive and IL-10 negative cells. Next, we studied whether the observed phenomenon was restricted to TLR4 signaling only by testing a wider variety of stimuli. We stimulated macrophages with the synthetic TLR1/2-agonist PAM3CSK4, TLR3-agonist Poly (I:C), TLR7/8-agonist R848 and TLR9-agonist CpG-C. Additionally, the cytokines IL-4 and IL-13 were used to mimic an anti-inflammatory phenotype and recombinant IL-10 was used as a positive control. Interestingly, the same secretion profile with a similar fraction of produced IL-10 upon was observed upon TLR7/8 stimulation. For TLR1/2 stimulation also IL-10 secretion was observed, but TNFα to a lesser extent. The other TLR agonists did not yield any activation (**Supplementary Figure 3**). These results suggest that the observed heterogeneity in IL-10 production appears to be a predetermined cell-fate which is only initiated by specific pathways and is not affected by activation signal strength.

### Transcriptional profiling does not reveal a deterministic LPS-induced IL-10 producing subset

**Figure 2:**
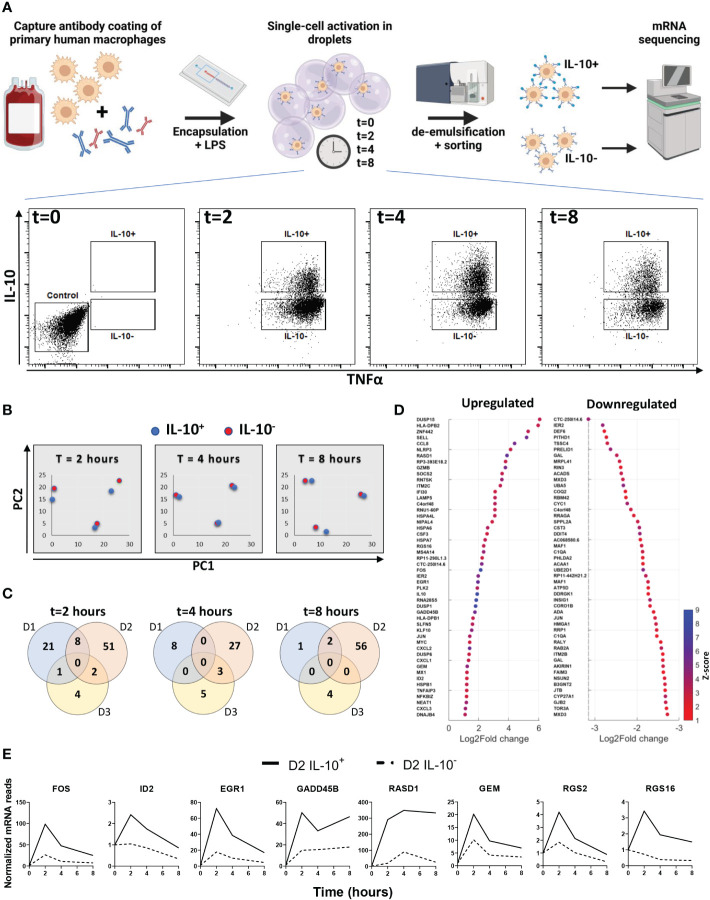
Transcriptional analysis of IL-10 positive and IL-10 negative macrophages. **(A)** experimental workflow; primary macrophages are coated with cytokine capture antibody and encapsulated in droplets with LPS. After 0, 2, 4 and 8 hours of stimulation cells are sorted into IL-10 producing and IL-10 non-producing populations. From each population mRNA is isolated and sequenced. **(B)** Principal component plot of IL10+ (blue dots) and IL10- (red dots) condition for all three timepoints and all three donors separate. **(C)** Venn diagrams showing the number of differentially upregulated genes per donor for every timepoint. **(D)** Top 50 log2fold upregulated differentially expressed genes for all donors over all timepoints. **(E)** Genes of interest upregulated in the IL-10+ sorted cells of donor 2 as expressed over time.

To test if the difference in secretion is determined by divergent signaling pathways or dedicated subsets, we set out to detect transcriptional differences between the two populations. Here, cells were stimulated in droplets with LPS for 0, 2, 4 and 8 hours and sorted into IL-10 producers (IL-10^+^) and IL-10 non-producers (IL-10^-^), followed by cell lysis, sequencing of mRNA content, and mRNA expression analysis (**Figure 2A**, **Supplementary Figure 4A**). Comparing both sorted populations to the unstimulated control indicated that LPS activation resulted in differential upregulation of a wide range of genes (**Supplementary Figure 4B**). Within this list of genes various characteristic pro-inflammatory genes were upregulated over the entire time course (**Supplementary Figure 4C**) (3, 53–56), confirming that LPS activation was successful. Thereafter, we analyzed the IL-10 mRNA expression in individual donors for the IL-10^+^ and IL-10^-^ populations. The three donors displayed two different trends of IL-10 mRNA expression in the IL-10^+^ cells (**Supplementary Figure 4D**), either a quick increase in the first two hours (donor 1 and 3), or a slow increase over 8 hours (donor 2). The first trend was displayed by two female donors of adolescent age, and the second trend was displayed by a middle-aged male donor, which is interesting as age and sex are known to affect inflammatory responses (57, 58). Although individually analyzed genes that are involved in the pathway between stimulation of TLR4 and activation of IL-10 showed no significant differential expression, IL10 mRNA expression was consistently around 2-3 times higher in IL10^+^ cells compared to IL10^-^ cells and unstimulated controls (**Supplementary Figure 5**) (59). A principal component analysis was performed on the IL-10 producers and non-producers of all three donors to compare the two different phenotypes (**Figure 2B**). Here, the IL-10^+^ and IL-10^-^ population clustered closely, whilst donor variation gave the most separation between samples, which was observed at all timepoints. This observation readily suggests that no big common differences were present between the two conditions, that bridged across all donors. This was further substantiated by examining overlapping differentially expressed genes between donors at all timepoints (**Figure 2C**). As no overlapping differences between donors were found, we examined the total list of differentially upregulated and downregulated genes for all donors individually at all timepoints (**Figure 2D**). Interestingly, despite not being differentially regulated in all donors we did find several genes that sparked our interest which had a distinct temporal expression in donor 2 (**Figure 2E**). FOS mRNA was found to be upregulated, which codes for a subunit of the AP-1 transcription factor that is shown to be responsible for IL-10 secretion (60–62). Additionally, EGR1 (63, 64) and ID2 (65, 66) have also been shown to be correlated with IL-10 secretion, and GADD45β (67) plays a role in anti-inflammatory behavior of macrophages. Interestingly, RASD1, GEM, RGS2, and RGS16 are all correlated to G protein-coupled receptor signaling. Although this is a very common signaling pathway with a wide variety of receptors, it might provide a clue on how the IL-10^+^ population is engaged, especially since AP-1 is shown to be upregulated in response to the cAMP transduction pathway, which is downstream of G protein receptor signaling (68). Nevertheless, no clear underlying mechanism or subset was found in the transcriptional profile of IL-10 producers. Moreover, to rule out the role of epigenetics we inhibited a range of histone deacetylases with the commonly used Trichostatin A, which did not alter the fraction of IL-10 producing cells (**Supplementary Figure 4E**). Taken together, we believe that the difference might be readily present before stimulation and thus missed using our approach, or that the combination of sorting and our sequencing methodology is not sensitive enough.

### Isolated cells produce a fraction of the amount of IL-10 that cells produce in bulk

**Figure 3:**
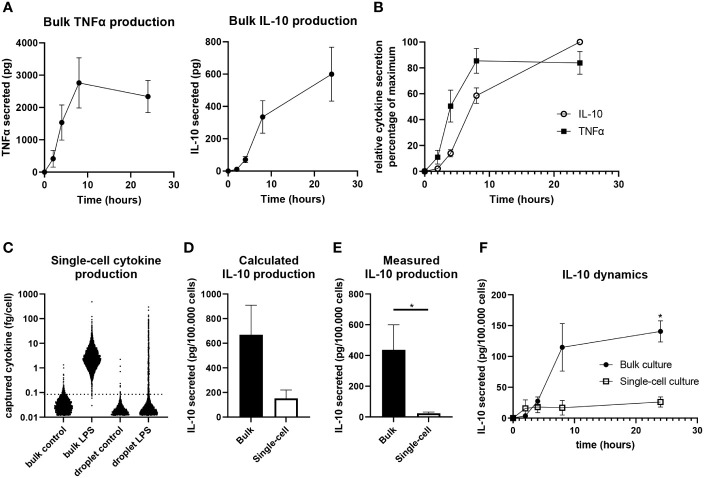
Quantification of IL-10 production in single-cell and bulk cultures. **(A)** Quantification of TNFα (n=5 independent donors) and IL-10 (n=9 independent donors) in bulk after LPS stimulation. **(B)** Relative secretion of TNFα (n=5 independent donors) and IL-10 (n=9 independent donors) as normalized to maximum per donor. **(C)** Quantified IL-10 production as calculated for individual cells of one representative donor using the titration curve of IL-10. **(D)** Total IL-10 production after 24 hours in bulk and single-cell culture as calculated using the IL-10 titration curve (n=3 independent donors). **(E)** Quantification of IL-10 production by bulk and single-cell LPS activated cells as measured using ELISA after 24 hours (n=7 independent donors), significance was tested *via* a paired t test with * indicating p<0.05 F) IL-10 production over 24 hours in bulk and single-cell culture as measured using ELISA (n=3 independent donors). Significance was tested using two-way RM ANOVA, and a *post-hoc* Šídák test with * indicating p<0.05.

To probe the secretion dynamics of LPS-stimulated macrophages, IL-10 and TNFα production in bulk was quantified by ELISA. Directly after activation TNFα is produced in large quantities for the first 8 hours (**Figure 3A**). However, between 8 and 24 hours no additional TNFα is produced and the total amount of TNFα is decreasing, which can be due to clearance/consumption (31) or IL-10-mediated inhibition (17, 20, 22). IL-10 production has a slower start but remains to be produced gradually between 2 and 24 hours after activation (**Figure 3A**). Comparing relative expression of both cytokines indicated that IL-10 dynamics closely followed the production of TNFα within about two hours (**Figure 3B**). Additionally, when the IL-10 production rate is picking up between 4 and 8 hours the TNFα production rate is starting to decline, strongly implying that a negative-feedback mechanism is in place.

Next, we aimed to quantify single-cell IL-10 secretion to allow comparison between bulk cultures and single-cell cultures, as we believe this is pivotal in understanding how macrophage heterogeneity translates to population wide control of inflammation. Therefore, we generated a titration curve to correlate measured single-cell fluorescent intensity after cytokine capture to the amount of cytokines captured (**Supplementary Figure 6**). This approach was an adaption of the technique described by Liu et al. (69) and enabled cytokine quantification in bulk and single-cell using the same method. This allowed the end-point calculation of captured cytokines per cell (**Figure 3C**), of which the sum showed that macrophages produce a much larger amount of IL-10 in a bulk situation compared to single-cell (**Figure 3D**). As this approach is limited to the capacity of capture antibodies and might suffer from inaccuracies due to diffusion of cytokines and capture reagents (52, 70), a second quantification approach was utilized. Cells were also activated in droplets and bulk without capture antibody coating to allow produced factors to remain in suspension for media sample collection after de-emulsification. After correcting for sample volume and cell number this allowed the direct comparison of IL-10 present in medium after bulk and single-cell stimulation as measured using ELISA. Slightly lower overall quantities were measured compared to the first method, but similar differences were observed between bulk and single-cells confirming much less IL-10 is produced in the absence of cellular interactions (**Figure 3E**). Additionally, we performed sampling over time which revealed that single-cells produce most of the IL-10 within the first 4 hours after stimulation, whereas bulk production continues for 24 hours, with the highest rate of production between the 4- and 8-hour timepoints (**Figure 3F**). This showed that isolated cells produce IL-10 in a short burst only whilst in bulk production is maintained over time. These results provide strong evidence that intercellular communication plays a dominant role in duration and speed of total IL-10 production.

### Paracrine signaling partially controls IL-10 production in macrophages

**Figure 4:**
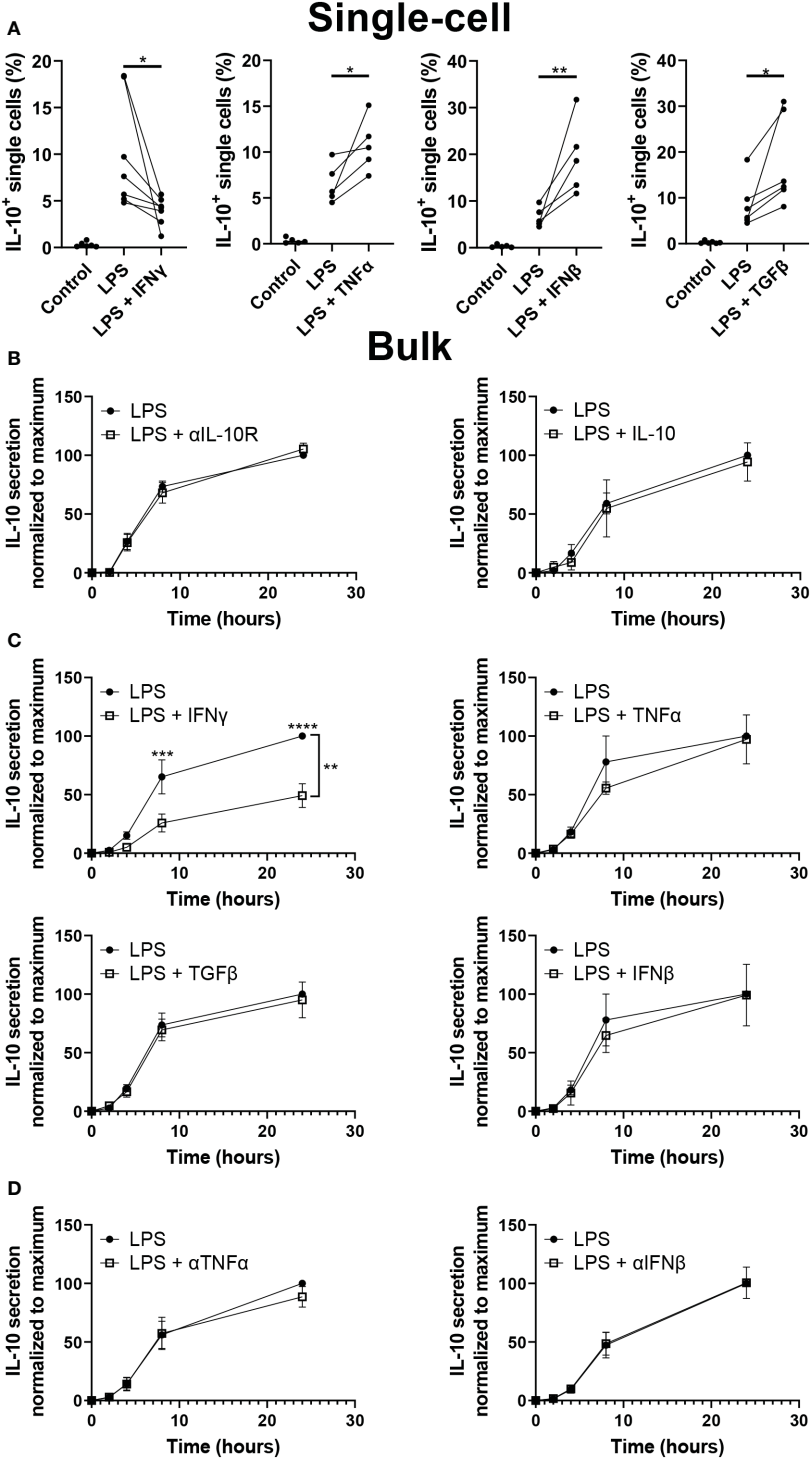
Effect of paracrine signals on IL-10 production. **(A)** Percentage of cells producing IL-10 after single-cell stimulation with LPS with or without IFNy (n=7), IFNβ (n=5), TNFα (n =5) or TGFβ (n=6). Significance was tested using One-way ANOVA and Tukey *post hoc* test. With * indicating p<0.05, and ** indicating p<0.01 **(B)** IL-10 quantification over 24h for bulk activated macrophages with and without IL-10R blocking antibodies (n=5 biological replicates) and with and without 100 pg IL-10 added 30 minutes prior to LPS activation. In the condition with IL-10 added, 100 pg of IL-10 was subtracted from final readout (n=3 biological replicates). **(C)** IL-10 quantification over 24h for bulk activated macrophages with and without IFNγ (n=5), TNFα (n=3), TGFβ (n=6), or IFNβ (n=3). Significance was tested using two-way RM ANOVA with ** indicating p< 0.01 for the variance attributed to treatment, and a *post-hoc* Šídák test with *** indicating p<0.001 and **** indicating p<0.0001. **(D)** IL-10 quantification over 24h for bulk activated macrophages with and without neutralizing antibodies for TNFα (n=4) or IFNβ (n=3).

As IL-10 quantification after LPS stimulation indicated an important role for paracrine signaling, we aimed to test several important regulators of macrophage secretion dynamics. To test their effect on IL-10 decision making interferon gamma (IFNγ), TNFα, interferon beta (IFNβ), and transforming growth factor beta (TGFβ) were added in droplets alongside LPS (**Figure 4A**). Literature describes that IFNγ is potently secreted in the inflammatory environment by T cells and macrophages themselves which results in activation of macrophages and progression of inflammation (23, 71, 72), additionally, it is commonly used to obtain a persistent M1 phenotype *in vitro (* 73). Our observations were in line with this as the fraction of IL-10 producers after LPS stimulation was significantly decreased when IFNγ was present. Although the effect of the pro-inflammatory TNFα and IFNβ is counterintuitive, it has been proposed in literature that they play a role in single-cell macrophage decision-making towards IL-10 production (16). The anti-inflammatory cytokine TGFβ, known to play a synergistic role together with IL-10 (74), indeed positively regulated the number of IL-10 producing cells. This demonstrated that various paracrine signals influence and control macrophage decision-making to produce IL-10. Additionally, as IL-10 signaling has been described as self-propagating (75–77), IL-10 mediated IL-10 secretion was also tested. The addition of recombinant IL-10 is not compatible with our capture system, therefore in bulk both blocking of the IL-10 receptor as well as priming of cells with recombinant IL-10 was tested during LPS stimulation (**Figure 4B**). However, no significant difference could be observed compared to the condition with LPS stimulation alone, showing that IL-10 does not self-regulate in this model. Furthermore, we performed bulk experiments with IFNγ, TNFα, IFNβ, and TGFβ as well, to investigate how the altered fractions of IL-10 producing cells translated from the isolated single-cell environment to the noisy bulk environment (**Figure 4C**). Interestingly, only the inhibitory effect of IFNγ could still significantly be observed, whilst the up-regulating effect of TNFα, IFNβ, and TGFβ was completely abolished, suggesting it was overruled by other signaling molecules or a different unknown parameter. To exclude the possibility that cells in these cultures were already producing at a maximum capacity, TNFα and IFNβ signaling was blocked using neutralizing antibodies (**Figure 4D**). However, this did not affect the IL-10 production either, indicating that these factors play a role in IL-10 production but are not key regulators. These results imply that cellular decision-making to produce IL-10 can be regulated at a single-cell level by multiple signaling molecules. However, in bulk cultures the effect of most individual regulators is overruled, potentially by other factors and the strong innate drive of macrophages to maintain immunological balance (2). Interestingly, the fact that IFNγ was still potent in decreasing IL-10 production in a bulk culture suggests that macrophage-mediated immunological homeostasis is more easily pushed to the inflammatory side, and thus, favors excessive inflammation over premature resolving of inflammation.

### Variable number of cellular interactions does not affect IL-10 production at small scale

**Figure 5:**
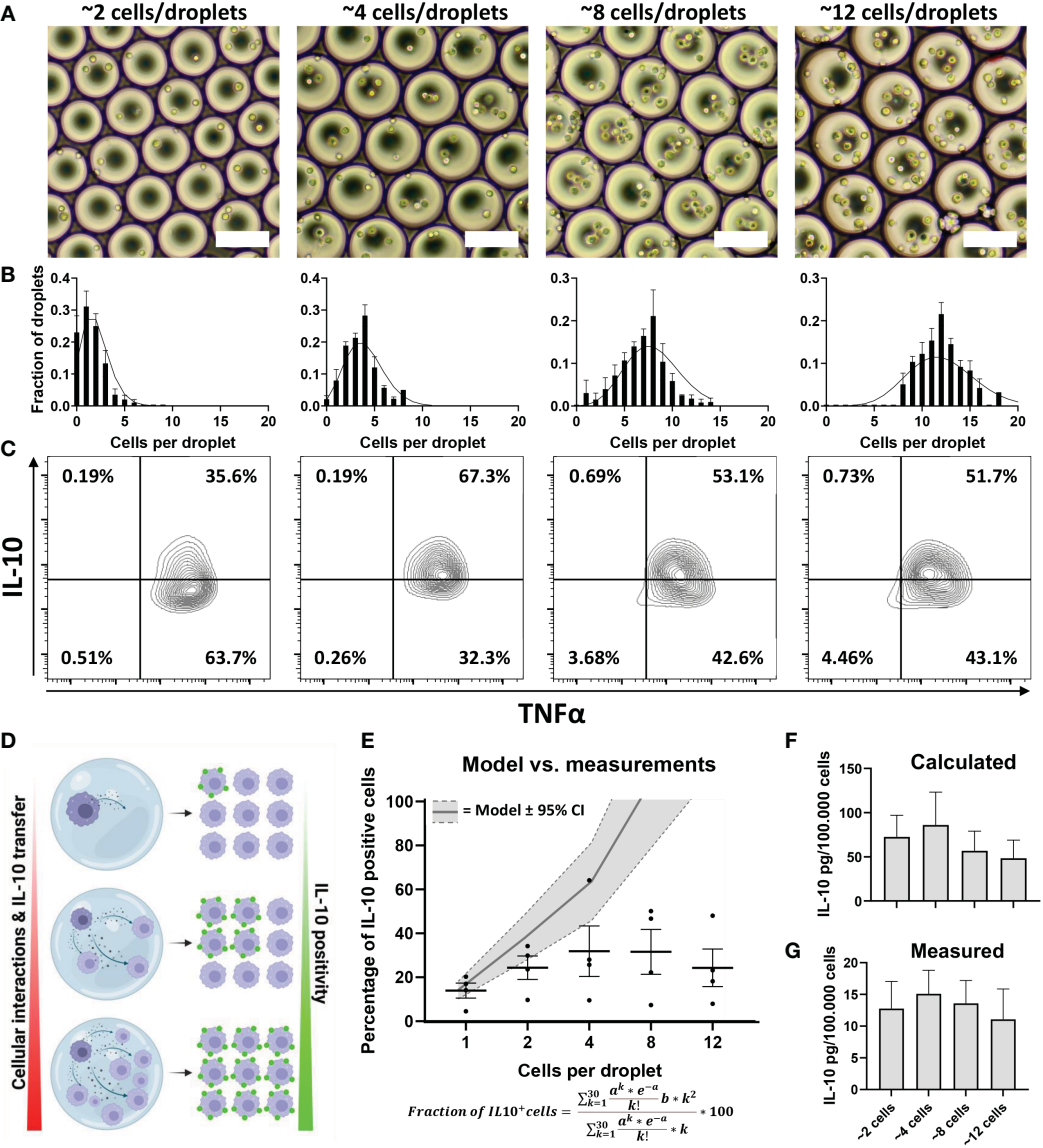
Effect of cellular interactions in droplets on IL-10 secretion. **(A)** brightfield microscopy images of droplets containing increasing numbers of cells. **(B)** Poisson distribution corresponding to the same concentrations (black lines) and the manually counted cell distribution of 50 droplets from 3 independent experiments. **(C)** Flow cytometric analysis of TNFα and IL-10 positivity of cells stimulated in multi-cell droplets with LPS. **(D)** Hypothesis of how increased cell numbers lead to increased cell positivity; as more cells are co-encapsulated, more cell interaction will take place, increasing the total amount of IL-10 produced but also more IL-10 transfers from producers to non-producers resulting in more positive cells. **(E)** Modelled prediction of increase in IL-10 positive cells based on percentage of producers in the single-cell experiments, width of grey band depicts 95% confidence interval. Measured percentage of cells becoming IL-10 positive in each condition are depicted as individual values and mean with SEM (n=4 biological replicates). **(F)** Calculated total amount of IL-10 produced based on single-cell fluorescent values (n=4 biological replicates). **(G)** Total amount of IL-10 secreted in droplets as measured using ELISA (n=5 biological replicates).

The previous experiments revealed that both the quantity of IL-10 production (**Figure 3**) and the fraction of producers (**Figure 4C**) are regulated by cellular signaling. To test the potency of this effect we produced larger droplets encapsulating varying numbers of macrophages (**Figure 5A**), allowing us to tune the number of cells participating in communication. We optimized cell concentration in these experiments so that droplets contained on average around 2, 4, 8 or 12 cells per droplet. Cell encapsulation occurs randomly and follows a Poisson distribution based on cell concentrations (78). Analysis of microscopy images confirmed that in our multi-cell droplet platform cell encapsulation followed the predicted Poisson distribution accurately (**Figure 5B**). After stimulating macrophages with LPS in these conditions and de-emulsification of droplets, cells were measured for IL-10 and TNFα secretion (**Figure 5C**). We hypothesized that increased numbers of cells encapsulated would result in increased fraction of IL-10 positive cells (**Figure 5D**), due to both increased cell interactions as well as produced IL-10 diffusing from one cell to the other, which was observed in bulk activation by us (**Supplementary Figure 2**) and others (70). We described this increase by a model which predicts the fraction of positive cells based on Poisson statistics, for cell contents up to 30 cells per droplet, and the averaged measured fraction of IL-10 secreting cells (**Figure 5E**). Even though the model predicted IL-10 positivity would increase and reach maximum, this did not fit the measured values. Although an increase could be observed between the first three conditions, an equilibrium was apparent for the higher cell-count conditions. To verify that a limit of IL-10 production was reached we used the previously described quantification methods (**Figure 3**) to verify these observations, which indeed showed minimal variation in response to increased cell interactions (**Figures 5F, G**). Additionally, cell viability appeared unaltered, ruling out a possible effect on secretory activity (**Supplementary Figure 7**). Although these results agree with the observation that cellular interactions partially control IL-10 production, they also indicate that this is not a strictly linear correlation. The fact that from a certain cell number a limit appears to be reached suggests that an additional factor comes into play, which limits IL-10 production.

### Cell density dictates macrophage inflammatory homeostasis through speed and duration of cytokine production

**Figure 6:**
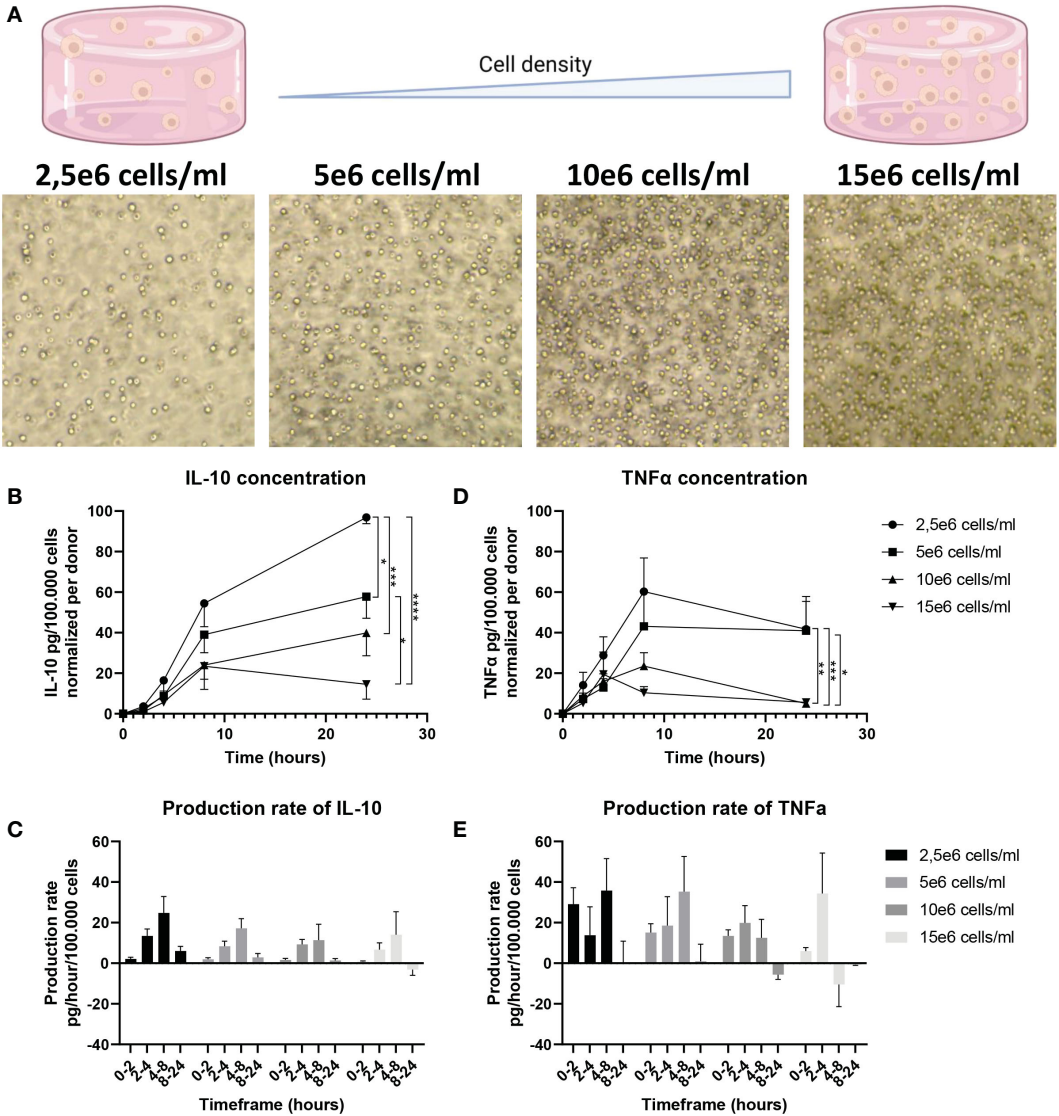
Effect of cell density on IL-10 secretion in 3D bulk cultures. **(A)** Brightfield microscopy images of cells at different densities in an agarose hydrogel. **(B)** IL-10 production over 24h quantified using ELISA for all 4 cell densities. IL-10 was normalized per donor to 100.000 cells. (n=7 biological replicates). Significance was tested using two-way RM ANOVA, and a *post-hoc* Šídák test with *p<0.05 **p<0.01, ***p<0.001, and ****p<0.0001. **(C)** Production rate of IL-10 per hour for all time intervals of all 7 donors. Normalized per donor to 100.000 cells. **(D)** TNFα production over 24h quantified using ELISA for all 4 cell densities. TNFα was normalized per donor to 100.000 cells. (n=5 biological replicates). Significance was tested using two-way RM ANOVA, and a *post-hoc* Šídák test with *p<0.05 **p<0.01, and ***p<0.001. **(E)** Production rate of TNFα per hour for all time intervals of all 5 donors. Normalized per donor to 100.000 cells.

**Figure 7:**
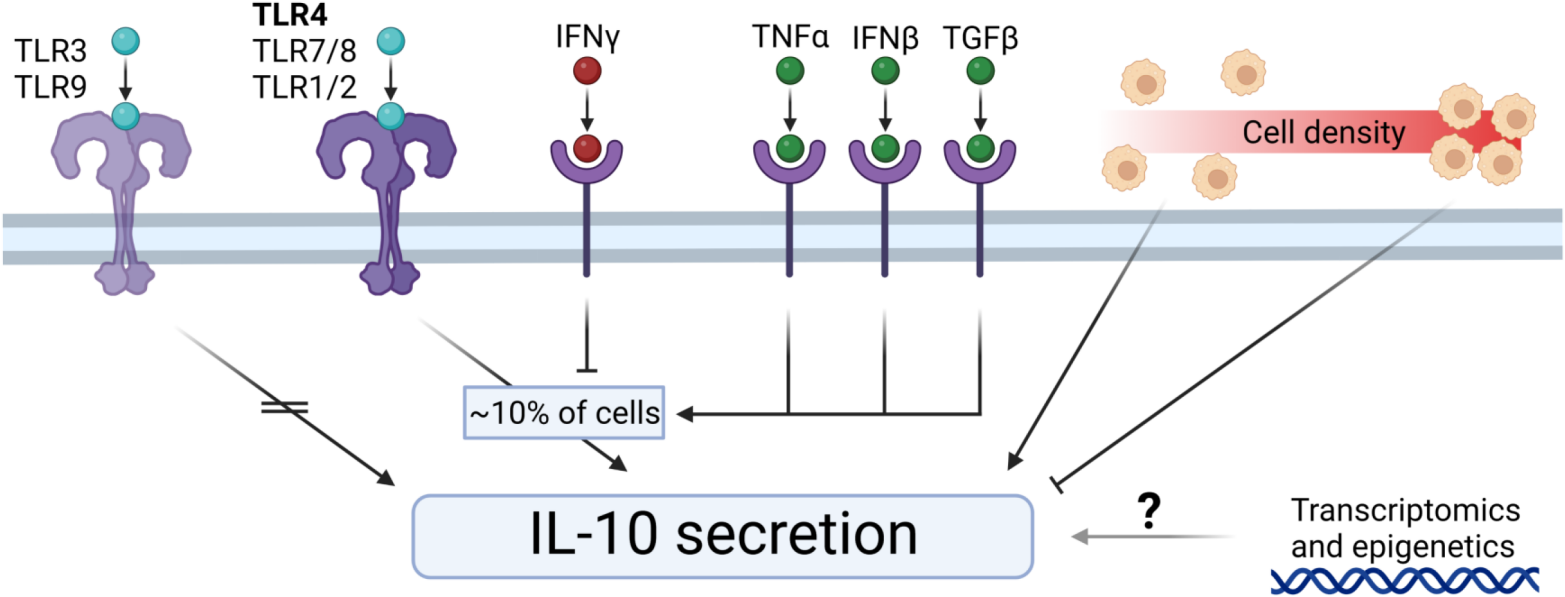
Extracellular control of IL-10 secretion by macrophages. TLR3 and TLR9 stimulation does not induce IL-10 secretion. TLR4, TLR7/8, and TLR1/2 initiates IL-10 secretion by a percentage of cells. IFNγ downregulates the fraction of IL-10 producers. TNFα, IFNβ, and TGFβ upregulate the fraction of IL-10 producers. Cell density regulates IL-10 secretion dynamics, where low-density results in relatively more IL-10 production, and high-density results in relatively less IL-10 production. The exact role of transcriptomics and epigenetics on the IL-10 secretion remains elusive.

The multi-cell droplet experiments were aimed at probing the effect of an increased number of cellular communications but in addition to cell count had cell density as a variable as well. To delineate the effect of cell density only, we translated this approach back to bulk cultures so cell count could be neglected and only cell density affects the cellular communication. Additionally, this allowed for temporal detection of secreted factors. According to recent literature varying density could result in an effect that is often coined “quorum sensing or quorum licensing” which influences cellular decision making on a population level (27, 79–81). To obtain a “tissue-like distribution” of macrophages, we trapped cells in agarose hydrogels, resulting in a homogenous 3D spatial distribution of cells whilst keeping concentrations identical to the multi-droplet experiments (**Figure 6A**). Cells were stimulated with LPS and over the course of 24 hours medium samples were collected to quantify TNFα and IL-10 as readouts of inflammatory homeostasis. Results showed that cells at lower density responded to LPS stimulation by producing a relatively higher total amount of IL-10 and producing IL-10 for the entire duration of the experiment (**Figure 6B**). Additionally, the highest production rate was observed in the lowest-density condition (**Figure 6C**), whereas the highest density condition had already stopped IL-10 production after 8 hours. The same effects were observed for TNFα, where the highest relative amount of TNFα was produced by the lowest two cell densities, and the highest cell-density had already stopped producing TNFα after 4 hours (**Figure 6D**). Additionally, the highest two cell densities displayed a negative production rate within 24 hours, suggesting active clearance of present TNFα (**Figure 6E**). These results indicate that macrophage-mediated inflammatory balance is strongly regulated by cell density. Not only does it show that total production is cell density dependent, but also that at higher cell densities, the return to inflammatory homeostasis is achieved much faster. This is very likely to be caused by reduced cell distance and reduced volume of media per cell, allowing faster paracrine signaling. Together with the other findings cell density can be incorporated into a small model describing the TLR signaling-mediated IL-10 secretion (**Figure 7**), in which, after TLR stimulation, the fraction of IL-10 producers can be altered by individual paracrine factors, but these mechanisms can be overruled in bulk by cellular communication mechanisms in which cell-density plays a major role by regulation of total IL-10 secretion.

## Discussion

Macrophages are commonly regarded as keepers of homeostasis (2, 82), by production and sensing of various cytokines. Therefore, their participation in secretory immune homeostasis at a local tissue level is widely accepted. We observed two different functional outcomes of seemingly phenotypically and transcriptionally identical populations at the single-cell level after LPS activation. This observation finds a resemblance with various occurrences in recent literature where a subset of cells in a population respond to perturbations and secrete signaling molecules to steer population behavior (18, 70, 83–86). This was elegantly described by Dueck et al. under the “crowd control” hypothesis, where heterogeneous responses by rare cells serve the purpose of balancing a rapid response to defend against pathogenic insult while avoiding self-toxicity (87). This hypothesis fits seamlessly with our observations of a small population of macrophages initiating anti-inflammatory behavior through IL-10 secretion. Therefore, we decided to investigate further how the observed phenomenon is regulated and could correlate to balancing of a pro- vs anti-inflammatory environment in a model of TNFα vs. IL-10 production. We observed that the functional heterogeneity to a certain extent was dependent on cellular communication at single-cell level, but that in bulk these effects were partially negated. Most likely, in bulk cultures the high degree of constant paracrine communication overruled all attempts of artificially altering the response towards increased anti-inflammatory feedback, indicating that macrophages are very capable of correcting for a single adjusted parameter in the pro-/anti-inflammatory signaling cascade. Interestingly, the reduction of IL-10 secreting cells by IFNγ translated to bulk cultures, suggesting that during infection homeostasis is more easily steered into excessive inflammation, rather than insufficient response. As the increase in the IL-10 fraction poorly translated to bulk, it led us to hypothesize that intercellular communication, correlated to cell number and cell density, is more potent in affecting the inflammatory imbalance than the underlying heterogeneity. Multi-cell droplet experiments indicated that IL-10 production was only to some extent correlated to the co-encapsulated number of cells, and that end-point measurement of IL-10 reached equilibrium at a certain cell number. Therefore, we switched to a bulk model that would consider both cell-density and secretion dynamics. When tested over time we indeed observed that increasing cell density, thus reducing intercellular distance and total media volume per cell, resulted in altered secretion dynamics for both IL-10 and TNFα. The prime difference was observed in the duration of the initial pro-inflammatory wave and the following response of anti-inflammatory cytokine production, which had a much faster fluctuation in the high cell-density populations. These findings fit the recent increase of literature discussing the effect of “quorum sensing/licensing” (79, 81) on macrophage inflammatory responses, where cell density and sensing of it, dictate the population wide responses (27, 80, 88). Various reports point towards a “master regulator” or “autoinducer” of such quorum sensing like TNFα or nitric oxide (28, 89), but those are not regarded here, as we observed that at the single-cell level several signals can skew the amount of IL-10 producing macrophages. Although that does not imply that such an autoinducer does not exist in the context of density-mediated control of inflammation, it does suggest that various secreted effectors are likely to play a role in anti-inflammatory decision making. Thus, a logical assumption is that the density-mediated effects we observed were likely due to the increased rate of paracrine interactions of all these effectors cumulatively, and the resulting secreted factors of the corresponding feedback mechanisms. Our findings once again underline the high degree of complexity that regulates how immune responses are initiated and balanced. Additionally, it highlights the importance for a further delineation of these immensely complex cell-signaling networks, and their role in tissue inflammatory homeostasis.

## Data availability statement

The raw sequencing data presented in the study are publicly available. This data can be found here: https://www.ebi.ac.uk/ena/browser/view/PRJEB59180.

## Author contributions

BT, ASm, and JT designed the study. BT, SH, and ASt performed all experiments. BT analyzed the data. BT and JT wrote the article. ASm and JT supervised the research and JT acquired funding. All authors contributed to the article and approved the submitted version.
