# Supplementary material for: Single-cell analysis reveals TLR-induced macrophage heterogeneity and quorum sensing dictate population wide anti-inflammatory feedback in response to LPS
Source: Front Immunol. 2023 Feb 24;14:1135223. doi: 10.3389/fimmu.2023.1135223 (PMC9998924; doi:10.3389/fimmu.2023.1135223)
Supplement: Supplementary file 1 [file DataSheet_1.pdf]

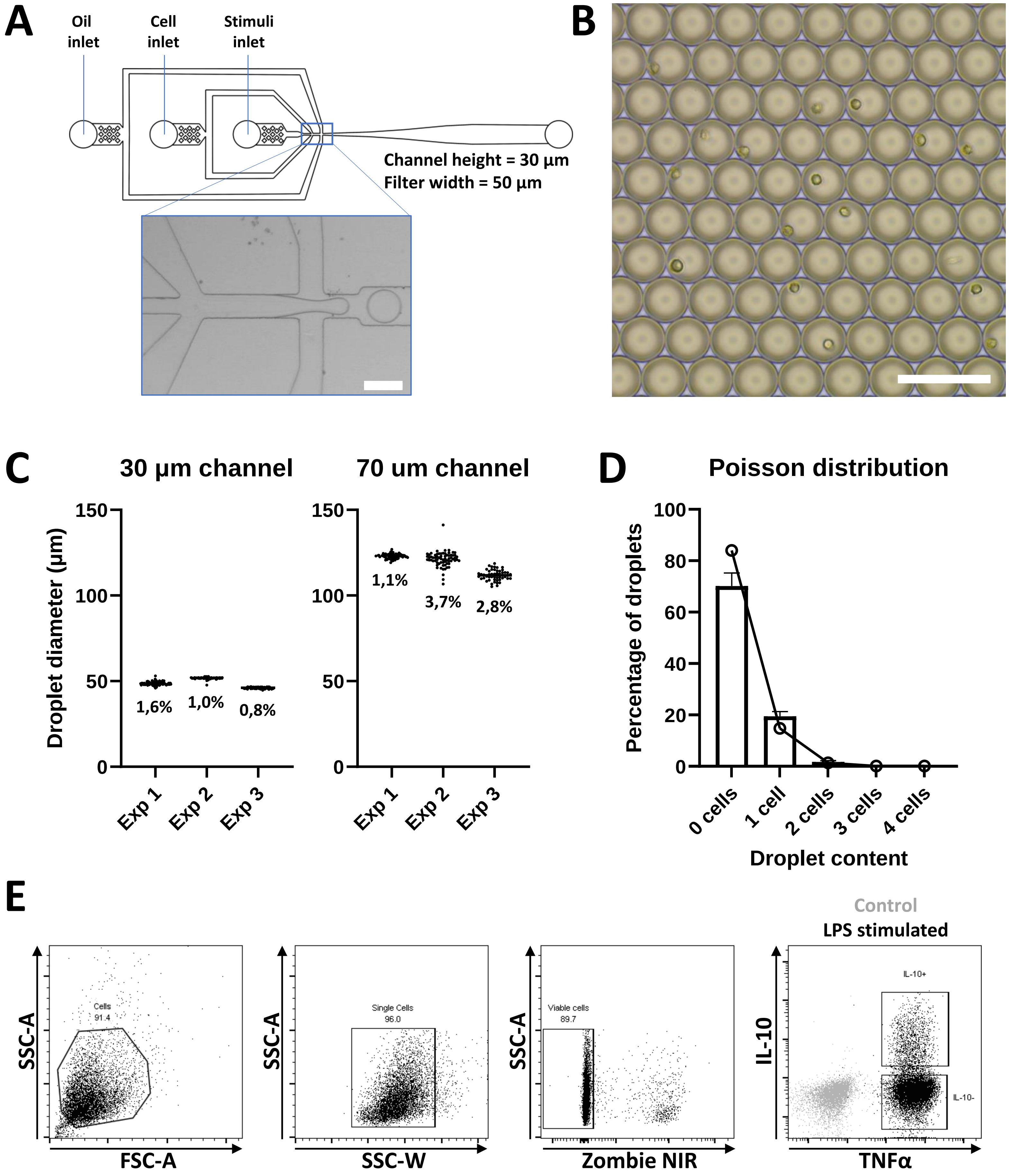

Supplementary figure 1: Droplet-based single-cell platform A) Layout of microfluidic droplet device. Scale bar represents 100 µm. B) light microscopy image of droplets containing single macrophages. Scale bar represents 100 µm. C) Droplet sizes as measured using “analyze particle” in ImageJ. Data shows 3 independent experiments for both devices with 30 and 70 µm channel height, percentages display coefficient of variance. D) Cell distribution among droplets as manually counted (bars) and predicted based on cell concentration (line). Data shows the content of 5 independent experiments with around 200 droplets counted each. E) Gating strategy from left to right; Cells are selected, single-cells are selected, viable cells are selected, IL-10^{+}/TNFα^{+} and IL-10^{-}/TNFα^{+} cells are gated in the LPS stimulated cells (black dots) with gating based on unstimulated control (grey dots). Data shows gating of cells from one representative donor.

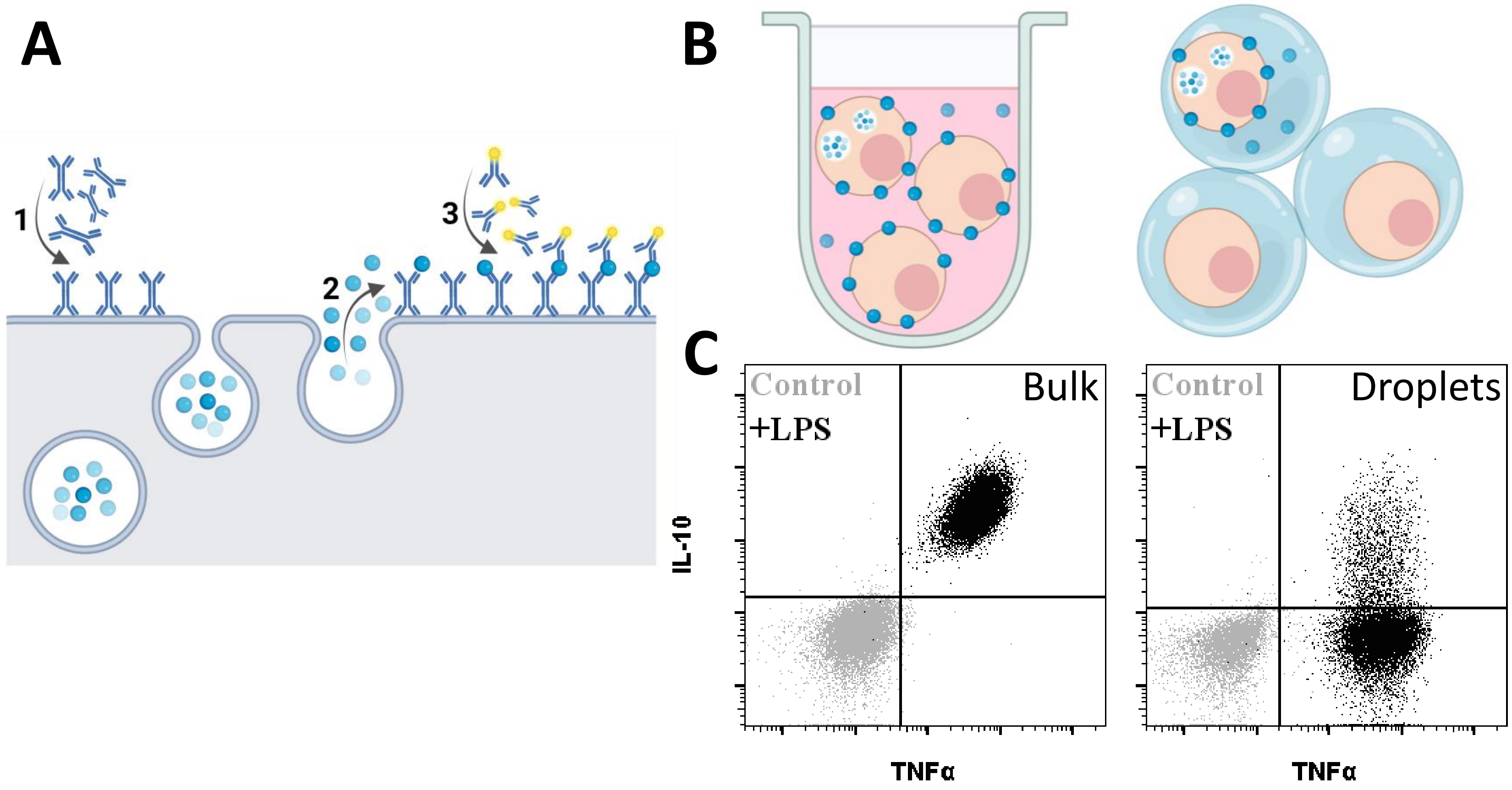

Supplementary figure 2: Cytokine capture assay. A) working principle of cytokine capture assay. Cells are coated with double antibodies (1), when cells secrete cytokines, these bind to the antibodies (2), these bound cytokines can then be detected using fluorescent antibodies (3). B) Difference between using cytokine capture in bulk culture and droplet culture. In bulk secreted cytokines from one cell can adhere to other cells, creating false positives, whilst in droplets cytokine capture is limited to only the producing cell. C) Flow cytometric results of IL-10 and TNFα detection on macrophages when stimulated in bulk and in droplets. Data shows results of one representative donor.

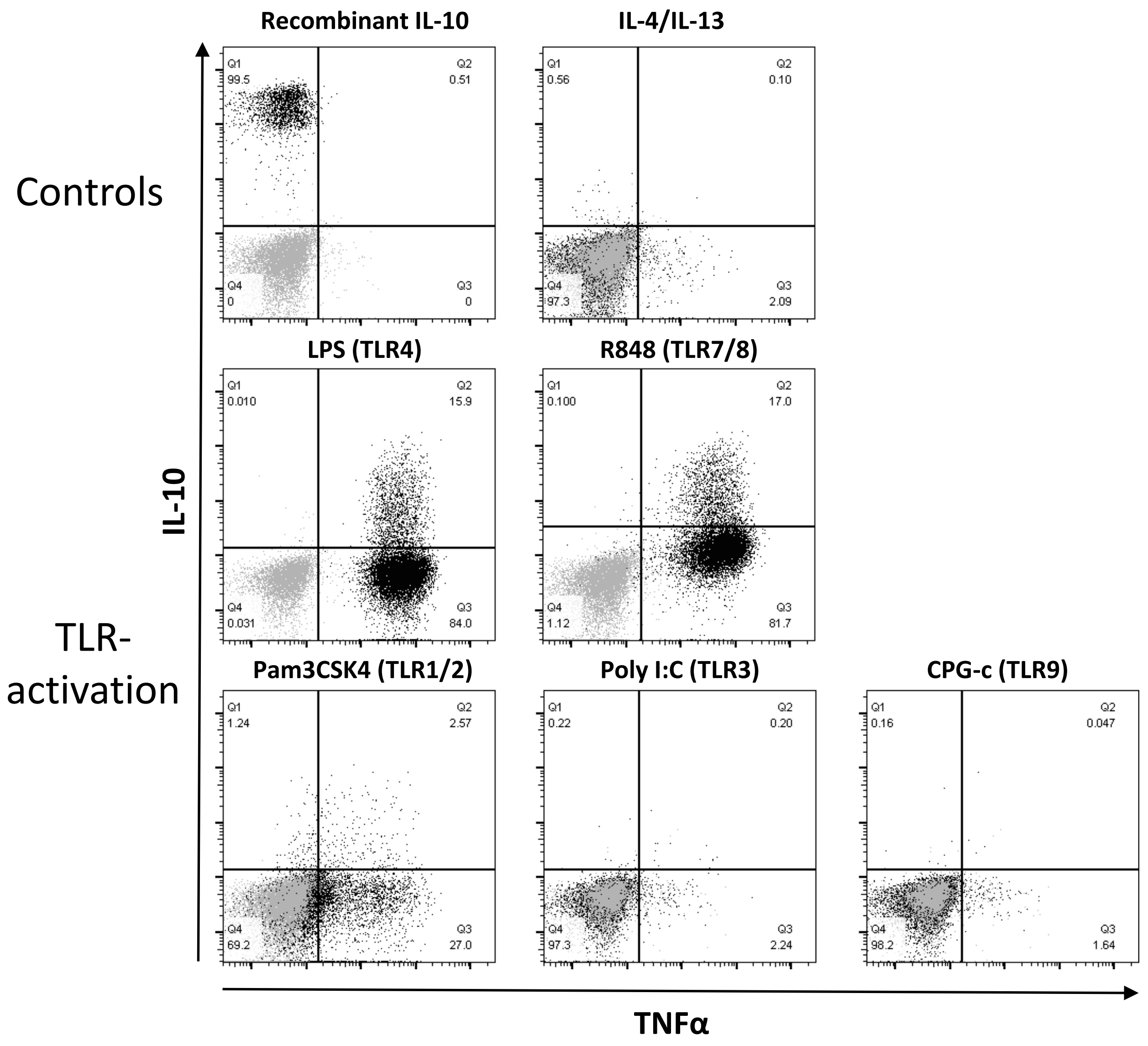

Supplementary figure 3: TNFα and IL-10 secretion by macrophages in response to various stimuli. Capture antibody coated macrophages were cultured in droplets for several control conditions and several Toll-like receptor agonists. TNFα and IL-10 secretion of viable cells was measured and depicted in dot plots. Grey dots represent unstimulated control, black dots represent added cytokines or stimuli. Data displays results from one donor.

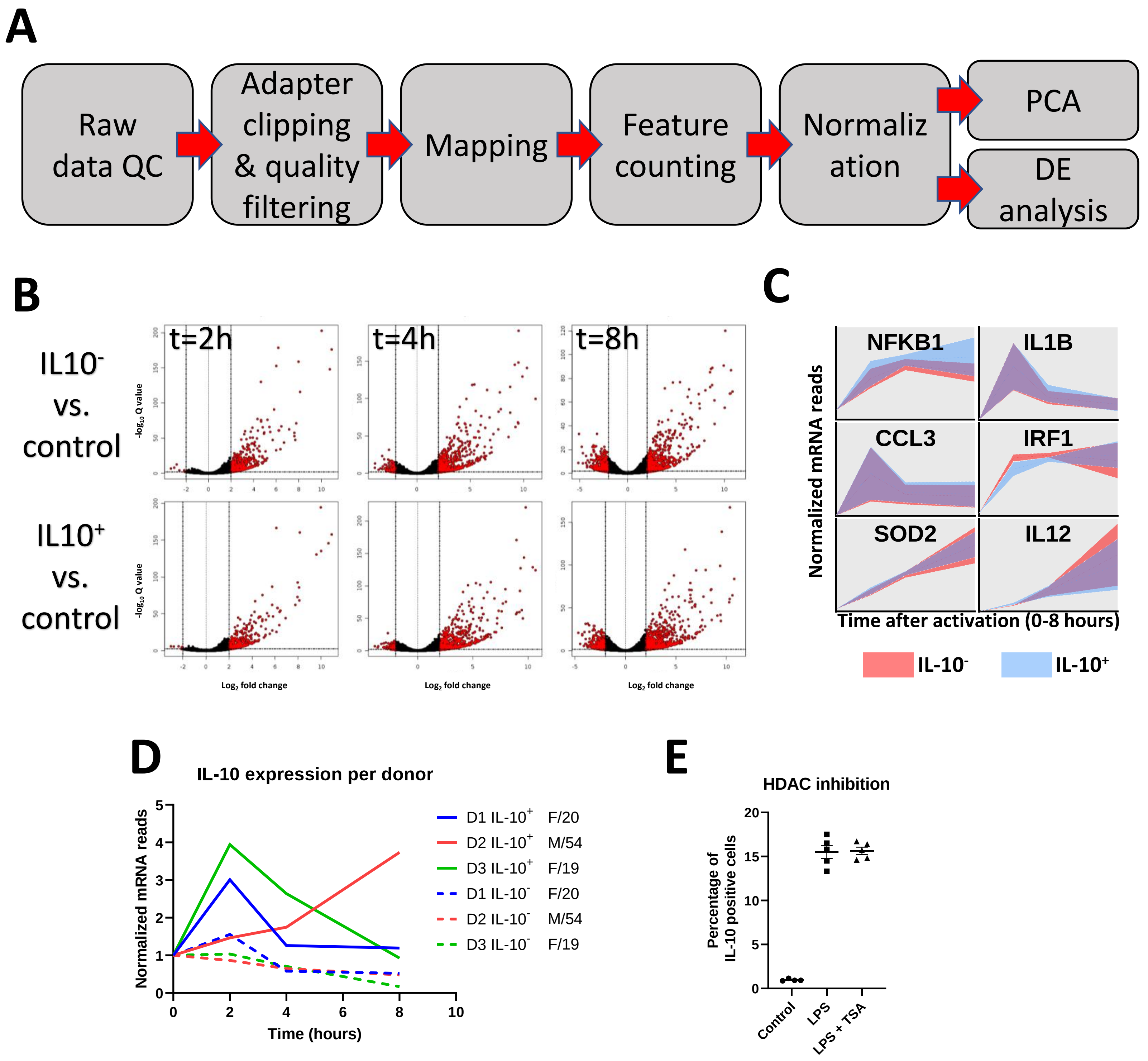

Supplementary figure 4: Sequencing analysis. A) Data analysis approach from raw data to analyzed results. B) Volcano plots of each timepoint of both the IL10+ and IL10- condition vs the control condition, representing 3 biological replicates, where red dots are significantly upregulated genes based on a p-value less than 0.05 and a log2 fold change higher or lower than 1 or -1, respectively. C) mRNA reads over time of common pro-inflammatory genes as upregulated in both IL10+ (blue) and IL10- (red) conditions by LPS stimulation. Band width represents SEM of 3 biological replicates and data is normalized to control condition. D) The different expressions of IL-10 mRNA over time for the three donors with sex (Female=F or Male=M) and age indicated. E) Result of HDAC inhibition by Trichostatin A, data shows results from n=5 independent donors.

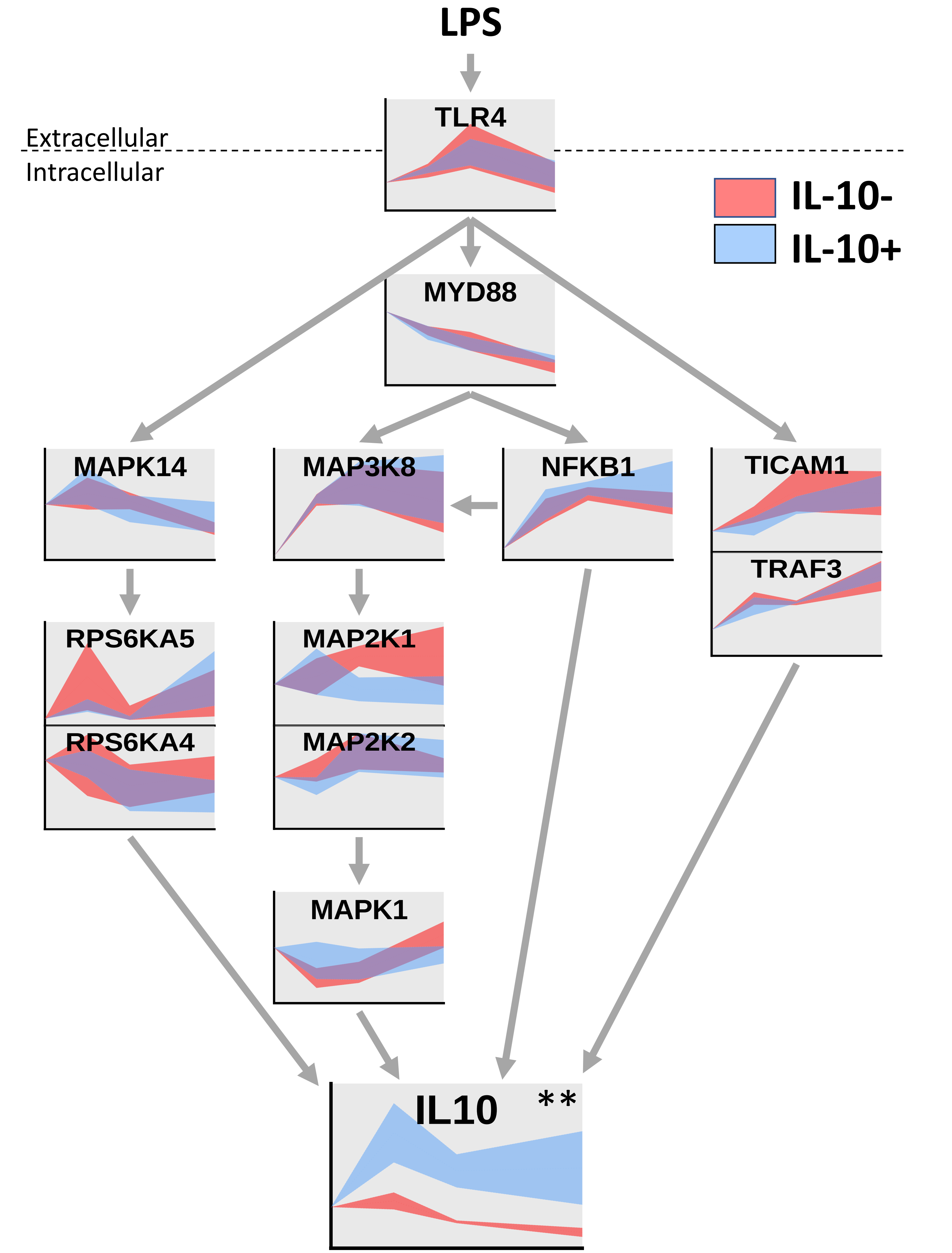

Supplementary figure 5: Gene expression of genes active in TLR4 activation/IL-10 secretion pathway. Expression of genes by IL-10 positive vs IL-10 negative cells after 2, 4 and 8 hours after LPS stimulation. mRNA reads are normalized within donors to unstimulated controls and depicted as red (IL-10 negative) or blue (IL-10 positive) bands where width represents SEM of n=3 independent donors. Significance testing was performed using two-way RM ANOVA, with ** indicating p< 0.01 for the variance attributed to Il-10 positivity, post-hoc analysis did not reveal significance at individual timepoints. (Figure adapted from Saraiva et al: http://dx.doi.org/10.1038/nri2711).

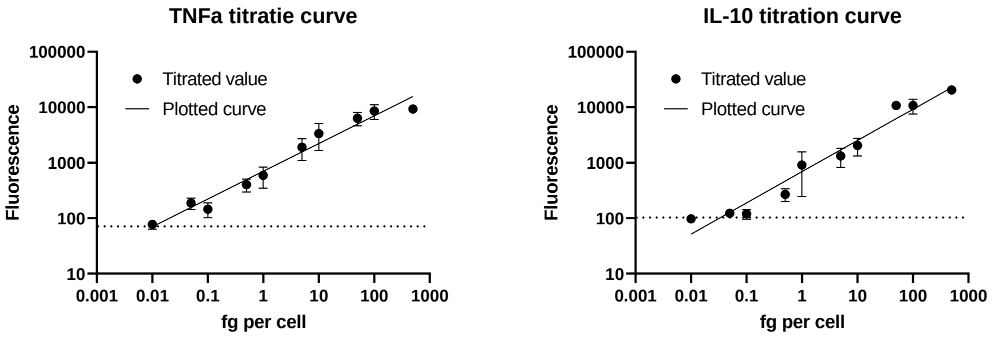

Supplementary figure 6: Titration of IL-10 and TNFα capture on cells and plotted curve to correlate single-cell fluorescent intensity to quantity of cell-bound cytokines. Data represents results from n=3 independent donors.

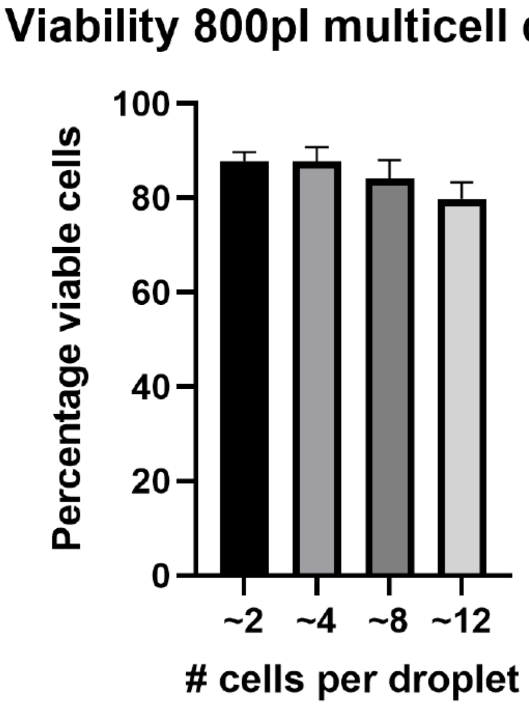

Supplementary figure 7: Cell-viability in multi-cell droplets. Viability of cells after multi-cell encapsulation based on Zombie NIR viability staining. Error bars represent SEM of 5 independent donors.
